# Assessing the Effect of Pretreatments on the Structure and Functionality of Microbial Communities for the Bioconversion of Microalgae to Biogas

**DOI:** 10.3389/fmicb.2018.01388

**Published:** 2018-06-26

**Authors:** Olivia Córdova, Rolando Chamy, Lorna Guerrero, Aminael Sánchez-Rodríguez

**Affiliations:** ^1^Laboratorio de Biotecnología Ambiental, Escuela de Ingeniería Bioquímica, Facultad de Ingeniería, Pontificia Universidad Católica de Valparaíso, Valparaíso, Chile; ^2^Department of Chemical and Environmental Engineering, Universidad Técnica Federico Santa, Valparaíso, Chile; ^3^Microbial Systems Ecology and Evolution, Department of Biological Sciences, Universidad Técnica Particular de Loja, Loja, Ecuador

**Keywords:** biogas, microalgae, *Chlorella*, methane, bioconversion, enzymatic pretreatment

## Abstract

Microalgae biomethanization is driven by anaerobic sludge associated microorganisms and is generally limited by the incomplete hydrolysis of the microalgae cell wall, which results in a low availability of microalgal biomass for the methanogenic community. The application of enzymatic pretreatments, e.g., with hydrolytic enzymes, is among the strategies used to work around the incomplete hydrolysis of the microalgae cell wall. Despite the proven efficacy of these pretreatments in increasing biomethanization, the changes that a given pretreatment may cause to the anaerobic sludge associated microorganisms during biomethanization are still unknown. This study evaluated the changes in the expression of the metatranscriptome of anaerobic sludge associated microorganisms during *Chlorella sorokiniana* biomethanization without pretreatment (WP) (control) and pretreated with commercial cellulase in order to increase the solubilization of the microalgal organic matter. Pretreated microalgal biomass experienced significant increases in biogas the production. The metatranscriptomic analysis of control samples showed functionally active microalgae cells, a bacterial community dominated by γ- and δ-proteobacteria, and a methanogenic community dominated by *Methanospirillum hungatei*. In contrast, pretreated samples were characterized by the absence of active microalgae cells and a bacteria population dominated by species of the Clostridia class. These differences are also related to the differential activation of metabolic pathways e.g., those associated with the degradation of organic matter during its biomethanization.

## Introduction

The advantages of using microalgae as substrate for biogas production (biomethanization) come from their biological and biochemical features, such as their ability to capture CO_2_ and use it to sustain growth, their high productivity in relation to other biomasses and to the possibility of converting all fractions of microalgae organic matter into biofuels (Sialve et al., 2009; González-Fernández et al., 2012; Bohutskyi and Bouwer, 2013). However, biofuels production from microalgae is not yet a system scalable to an industrially viable one (Zamalloa et al., 2011). This is mainly because microalgae cell wall is difficult to degrade by hydrolytic bacteria (such those commonly found in anaerobic sludge associated bacteria). Therefore, in the absence of available microalgae organic matter to feed the anaerobic digestion process, biogas production is deficient (González-Fernández et al., 2012).

One solution to this biotechnological problem is to apply a pretreatment to the microalgae cultures (Angelidaki and Batstone, 2010; Mendez et al., 2013; Passos et al., 2014) with the purpose of increasing the availability of soluble organic matter and thus improving biogas production yield (Bohutskyi and Bouwer, 2013). Different pretreatments may be applied: physical pretreatments (by applying a physical force and/or heat) or enzymatic pretreatments (by adding enzymatic raw extracts or commercial enzymes). Enzymatic pretreatments aim at increasing the selective permeability of the microalgae cell wall to release inner compounds as well as to solubilize cell wall constituents (González-Fernández et al., 2012; Mahdy et al., 2016). Enzymatic pretreatments change the way in which microalgae organic matter is made available in the medium and lead therefore to a new configuration of the microalgal biomass (respect to the organic matter configuration prior the pretreatment). The concept of biomass configuration will be used in this study to refer to the way in which organic matter from the microalgal biomass becomes available in the medium.

Bareither et al. (2013) characterized the microbial diversity (bacteria and archaea) during the biodegradation of urban solid waste in two conditions (solid waste and leachate of solid waste) and correlated it to methane production. Authors concluded that microbial communities were not similar between conditions. Their results support the hypothesis that the identity of functionally active species in anaerobic sludge associated microbial communities changes, not only for each substrate type, but also for the same substrate under different conditions. There are also studies that report changes in the structure, e.g., changes in the species diversity of microbial communities when using different substrates for biogas production under the same operating conditions in the laboratory (Lee et al., 2009; Kampmann et al., 2012). Aforementioned studies assessed changes in microbial communities during biogas production at a rather low resolution: it means without providing information on specific activated/repressed pathways across conditions (substrates and/or pretreatments). One could hypothesize that changes in the configuration of the microalgal biomass drive structural i.e., species being present, and also functional changes i.e. pathways being activated/repressed in the anaerobic sludge associated microbial communities during biomethanization. However, such hypothesis is poorly addressed in the literature.

Addressing changes in anaerobic sludge associated microbial communities during biogas production at a better resolution is now possible thanks to current developments of the so called “omic” technologies. The development of what has been termed “omic” techniques, particularly those that apply to genetic material isolated directly from environmental samples, i.e., metagenomic, metatranscriptomic, allows for the evaluation of structural (changes in the relative abundance of species), and functional dynamics (changes in genetic expression) of microbial communities (Jansson et al., 2012). Meta-omics studies have even been possible in the context of bioreactors, generating knowledge on how the configuration of a reactor and its operating conditions influence the microbial community (Zhang et al., 2010; Vanwonterghem et al., 2014). Meta-transcriptomic studies allow the identification of near full length transcripts being expressed by a given microbial community under a set of experimental conditions (Moset et al., 2015; Nolla-Ardèvol et al., 2015; Stolze et al., 2015). Such studies can be used to identify differentially expressed genes across conditions.

In the present study, we describe the impact that the enzymatic pretreatment of microalgal biomass has on the anaerobic sludge associated microbial communities during biogas production. Pretreatment impact is analyzed at the level of species composition as well as on what respect to the activation/repression of metabolic pathways. We did so by reconstructing the metatranscriptome of anaerobic sludge associated microbial communities during the biomethanization of *Chlorella sorokiniana*, with and without enzymatic pretreatment to increase the solubility of organic matter and to achieve significant increases in biogas production.

## Materials and methods

### Microalgae culture

A culture of *C. sorokiniana* (Shihira and Krauss, 1965) isolated from an effluent of anaerobic sludge digestors belonging to a waste water treatment plant in Spain, was donated by the University of Huelva, Spain. The Sueoka culture medium (Sueoka, 1960) was used to maintain this culture in the laboratory. The culture was grown on 5 L flasks under non-sterile conditions at a temperature of 21 ± 2°C, with artificial lighting of F24-39 W and I = 127.60 μmol of photons/(m^2^ × s), 24-h light photoperiod and aeration of 1.3–1.5 L/min of atmospheric air.

*Chlorella sorokiniana* biomass composition was characterized in what respect to total protein content by the Kjeldahl method which measures total organic nitrogen (Owusu-Apenten, 2002; Safi et al., 2013). Total lipids where determined by Soxhlet method (APHA-AWWA-WPCF, 1999), and carbohydrate by the Dubois method (Dubois et al., 1951). Recalcitrant material, measured as the insoluble fiber content of the sample, was determined using acid digestion followed by alkaline digestion (APHA-AWWA-WPCF, 1999).

### Enzymatic pretreatment

For enzymatic pretreatment application 400 mL of microalgal biomass was used at an enzyme/substrate ratio of 1%, pH 7 for 24 h at 37°C. The Ns22128 enzyme (cellulase) from Novozymes® was used for this purpose.

### Cell wall rupture

Microalgae cell wall rupture was evaluated by SYTOX Green staining in pretreated cells (Sato et al., 2004). This probe has a high affinity for nucleic acids and, only penetrate cells whose cell membranes are damaged. In this way, probe fluorescence and the microalgae autofluorescence were used to mark dead cells (due to rupture or damage) and live cells respectively.

### Biochemical methane potential (BMP)

Methane production from *C. sorokiniana* cultures was evaluated using a biochemical methane potential test (BMP) (Angelidaki et al., 2009). The inoculum used came from an anaerobic sludge reactor fed with sludge from a waste water treatment plant located at “La Farfana”, Santiago, Chile. Bottles of 100 mL capacity were used for the BMP test. All flasks were inoculated at 0.5 g. of volatile solids (VS) substrate/g. of VS inoculum ratio. Bottles containing only the inoculum were used as controls in order to correct for inoculum methane yields. We assessed the methane production from the inoculum, determined in blank assays with medium, and no microalgal biomass, which is subtracted from the methane production obtained with microalgal biomass assays. Enzyme control, biomass control, and inoculum control were performed for each BMP assay.

Bubbles were made in the bottles using a mix of gases (80% N and 20% CO_2_) in order to ensure anaerobic conditions, and were then sealed and kept at 37°C. The test ended once the methane production had stopped.

The percentage of CH_4_ in the biogas was determined by gas chromatography using a Perkin Elmer Clarus 500 chromatograph, oven temperature 80°C, detector TCD at 120°C, and injector 80°C. Helium was used as carrier with a Hayesep Column Q 4 m × 1/8″ OD (13 ft.). One milliliter of biogas was taken with a glass syringe and then injected into the port of the Gas Chromatograph. Determinations were performed by triplicates to estimate the average value of CH_4_ percentage present in the biogas.

CH_4_ production was quantified by displacement of a NaOH solution due to carbon dioxide absorption. The accumulated CH_4_ production in time (accumulated CH_4_ mL/g. VS of substrate) was normalized to mL/g. VS of substrate using Equation (1).

(1) $$\begin{array}{l} \frac{mL\;of\;CH_{4}}{g.\;VS\;of\;substrate}=\frac{mL\;of\;produced\;CH_{4}}{\frac{g.\;VS\;substrte}{L}\;\times \;mL\;of\;substrate\;in\;a\;bottle\;} \end{array}$$

### Methane productivity modeling

Methane productivity was modeled using the modified Gompertz model (Donoso-Bravo et al., 2010) based on the values observed during the BMP test according to Equation (2):

(2) $$\begin{array}{l} B=P\;\times \exp\left(-\exp\left(\frac{Rm\;\times \;e}{P}\left(\lambda -t\right)+1\right)\right) \end{array}$$

where *B* represents the accumulated volume of CH_4_ produced at time *t* (in days), *P* the maximum CH_4_ production potential (mL CH_4_/g. VS of substrate), *Rm* the maximum production rate (mL CH_4_/g. VS of substrate/day), λ the duration of the latency stage (in h), and *t* the incubation time (in days).

### Analytical methodology

All analyses were performed by triplicates and average values and their standard deviations estimated for every biological replicate during the BMP assay. Both microalgal biomass and inoculum were characterized according to standard methods (APHA-AWWA-WPCF, 1999) to quantify physical-chemical parameters such as: total solids (TS), VS, and volatile suspended solids (VSS). The pH was measured with a HI/111 Hanna Instrument pH meter with a sensitivity of ±1 mV, which corresponds to 0.01 units of pH.

### Statistical analysis

Statistical analyses were performed to determine if there were significant differences between the conditions with and without enzymatic pretreatment in relation to their BMP. The analyses were performed using the software Statistica 13 (StatSoft Inc., Tulsa, USA, 2016). Each time a variance analysis was used to check whether the normality and homoscedasticity assumptions had been met. When the assumptions had not been met, the data was properly transformed.

### RNA extraction and quantification for sequencing libraries

Samples from both experimental conditions were taken directly from the BMP test bottles. Samples were first centrifuged at 10,000 rpm for 10 min to discard the supernatant and the pellet was stored at −80°C until the moment of analysis. Total RNA extraction was performed using the PowerSoil RNA Isolation® (MOBIO) extraction kit following the manufacturer's instructions. RNA samples were then treated with the DNase Max Kit (MOBIO) to remove possible genomic DNA contamination. The samples were freeze dried for 6 h and send out for library preparation and Illumina sequencing at Molecular Research LP MRDNA Laboratory (Shallowater, Texas, USA). Total RNA concentration was determined using the Qubit® RNA Assay Kit (Life Technologies). RNA integrity value (RIN) was determined with the Agilent RNA 6000 Nano Reagents and RNA Nano Chips in Agilent 2100 Bioanalyzer (Agilent Technologies). Between 0.5 and 1.5 μg of the total RNA were used to remove the DNA using the Baseline-ZERO™ DNase (Epicentre) kit according to the manufacturer's instructions. In order to only retain the total mRNA fraction, the rRNA was eliminated from the RNA samples which were already free of DNA, using the Ribo-Zero™ Magnetic Gold Kit (Bacteria; Illumina). These samples were used library preparation with the TruSeq™ RNA LT Sample Preparation Kit (Illumina) according to the manufacturer's instructions. The final concentration of all libraries was measured using the Qubit® dsDNA HS Assay Kit (Life Technologies) and their average size was determined with an Agilent 2100 Bioanalyzer (Agilent Technologies). Libraries were pooled in an equimolar proportion of 2 nM, and 8 pM of the pool was pair-end sequenced for 300 cycles using a HiSeq 2500 system (Illumina).

### Metatranscriptome sequencing and gene expression differential analysis

Sequence quality control was performed with the FastQC toolkit (Andrews, 2010). Reads were subject to “reads trimming” before analysis, in order to eliminate stretches of low quality. Subsequently, a *de novo* metatranscriptome assembly was performed using the Trinity bioinformatics tool (Grabherr et al., 2011). Transcripts quantification was done using the RSEM tool, which implements an EM algorithm (Expectation Maximization) to maximize the verisimilitude that a fragment comes from a given transcript and then calculates digital values of gene expression (Li and Dewey, 2011). The detection of differentially expressed genes between the samples without pretreatment (WP) and with pretreatment (EP) was done using DESeq (Anders and Huber, 2010).

### Taxonomic analysis and functional annotation of microbial communities under conditions with/without enzyme pretreatment

Taxonomic annotations at the species level of each reconstructed transcript was done by BLAST (https://blast.ncbi.nlm.nih.gov/Blast.cgi). We also downloaded the sequence of the rRNA *16S* gene of the species for which at least one differentially expressed transcript was detected. These species were considered as “active species” in each of the experimental conditions.

### Prediction of activated key proteins/enzymes and of their corresponding metabolic pathways during anaerobic digestion

To identify metabolic pathways that were active at both experimental conditions (with and WP) during anaerobic digestion, the Kyoto Encyclopedia of Genes and Genomes-KEGG (http://www.genome.jp/kegg/) platform was used. We first mapped differentially expressed transcript to their corresponding genes in KEGG using the BlastKOALA tool. Genes where then mapped to metabolic pathways within KEGG. Finally, the XPathway was used based to compare metabolic pathways across experimental conditions. The XPathway is able to detect and quantify the metabolic differentiation between the two conditions (Temate-tiagueu et al., 2016).

## Results

### Microalgal culture characterization

The characterization of the *C. sorokiniana* biomass showed the following relative composition: 45.5% proteins, 26.2% lipids, 23.7% carbohydrates, and 4.70% of raw fiber (insoluble material) at pH 6.9. The concentration of TS (g/L) was 6.90 ± 0.10, VS (g/L) of 6.50 ± 0.10, and a Chemical Oxygen Demand (g/L) of 13.8 ± 1.30.

### Biochemical methane potential (BMP) and productivity

**Figure 1:**
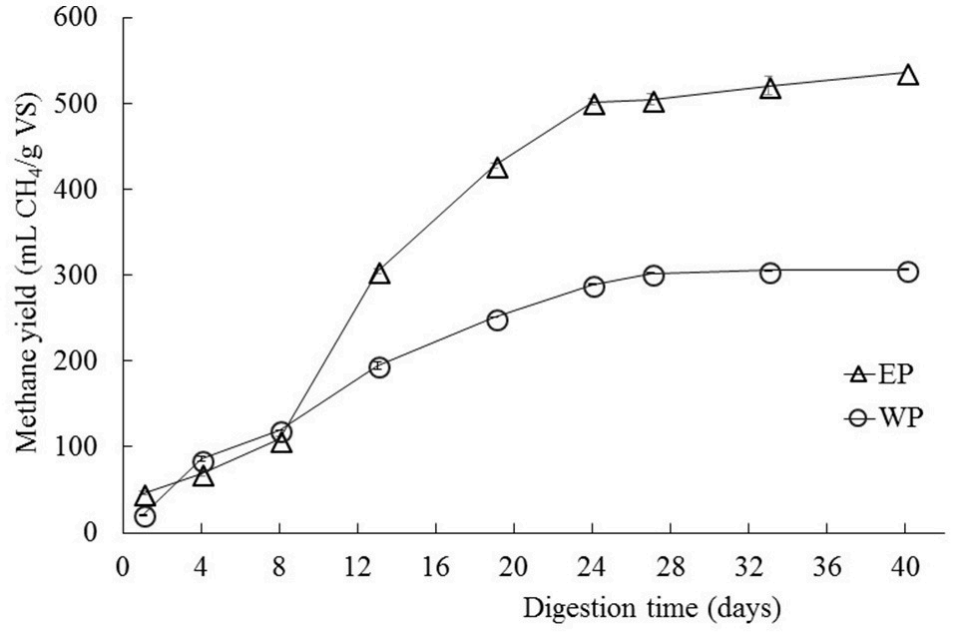
Biochemical methane potential of *C. sorokiniana* when subjected to enzymatic pretreatment (cellulase) under conditions of 1% enzyme-substrate concentration, pH 7, for 24 h (EP data series). The biochemical methane potential is also shown for control samples (WP data series) that were not treated enzymatically. All test was performed by triplicates. Average values ± standard deviations are plotted in each case.

The cellulase pretreated microalgal biomass showed a methane production of 537 mL of accumulated CH_4_/g. VS of substrate, thus achieving a 75% increase in relation to the non-pretreated biomass with 307 mL of accumulated CH_4_/g. VS of substrate (Figure 1). Additionally, differences were observed between experimental conditions in the behavior of the accumulated methane curves i.e., in the latency stage, the methane production rate slope and in the biochemical methane potential. According to the values inferred by the modified Gompertz model, the maximum methane production rate (*Rm*) in the pretreated biomass was on average 2.65 times that one observed in the non-pretreated biomass (Table 1).

**Table 1:** Methane productivity inferred from observed values by fitting a Gompertz Model.

**Pretreatment**	**Condition**	**λ (h)**	***R_*m*_* (mL CH_4_/g. VS/d)**	***P* (mL CH_4_/g. VS)**	***R*^2^**
Enzymatic with cellulase	1% e/s-pH 7–24 h	3.31 ± 0.24	30.67 ± 3.27	545.68 ± 42.70	0.991
Biomass without pretreatment	–	1.60 ± 0.24	11.56 ± 0.15	317.66 ± 0.90	0.965

*λ, latency period; Rm, maximum production rate; P, maximum CH_4_ production potential. R^2^ value for adjustment between observed and modeled values. e/s, enzyme-substrate ratio. All test performed by triplicates. Average values ± standard deviations are provided*.

The average methane percentage found in the produced biogas during the BMP test was of 66.7 ± 1.5% which falls within known methane percentages in biogas (60–70%).

### Molecular analysis of anaerobic sludge microbial communities

After quality control of the raw data (see section Materials and Methods for details), a total of 29,618,400 reads were retained. From those retained reads we were able to reconstruct a total of 96,193 transcripts from all samples. An average of 32,552 transcripts were reconstructed from control samples (without enzymatic pretreatment) while 38,921 was the average of transcripts reconstructed for samples with enzymatic pretreatment. A subset of 15,088 transcripts were only expressed in samples without enzymatic pretreatment while 11,686 transcripts showed an expression only in the samples with enzymatic pretreatment. From this information, 227 differentially expressed genes across conditions were detected. All sequencing data generated during this study can be accessed at the NCBI Trace Archive (study ID SRP139287).

### Taxonomic and functional annotations of differentially expressed genes across experimental conditions

Taxonomic and functional annotation was only performed for the 227 differentially expressed genes across experimental conditions (from here on we refer to control samples as WP and to samples with enzymatic pretreatment as EP ones). Differentially expressed transcripts can be seen as the functionally active fraction that better represent the species with the highest responsiveness to the experimental conditions being tested (with and without enzymatic pretreatment). The unanalyzed transcripts (non-differentially expressed) have a high probability of being part of constitutive metabolic pathways that are unrelated to the cellular responses triggered/repressed by the experimental conditions. Based on the above, it should be noted that the analysis of the relative abundance of differentially expressed transcripts allowed us to identify the structure of the functionally active microbial community by providing information on the existing taxa, as well as differentially active metabolic pathways between the conditions.

#### Functionally active taxa in control samples

**Figure 2:**
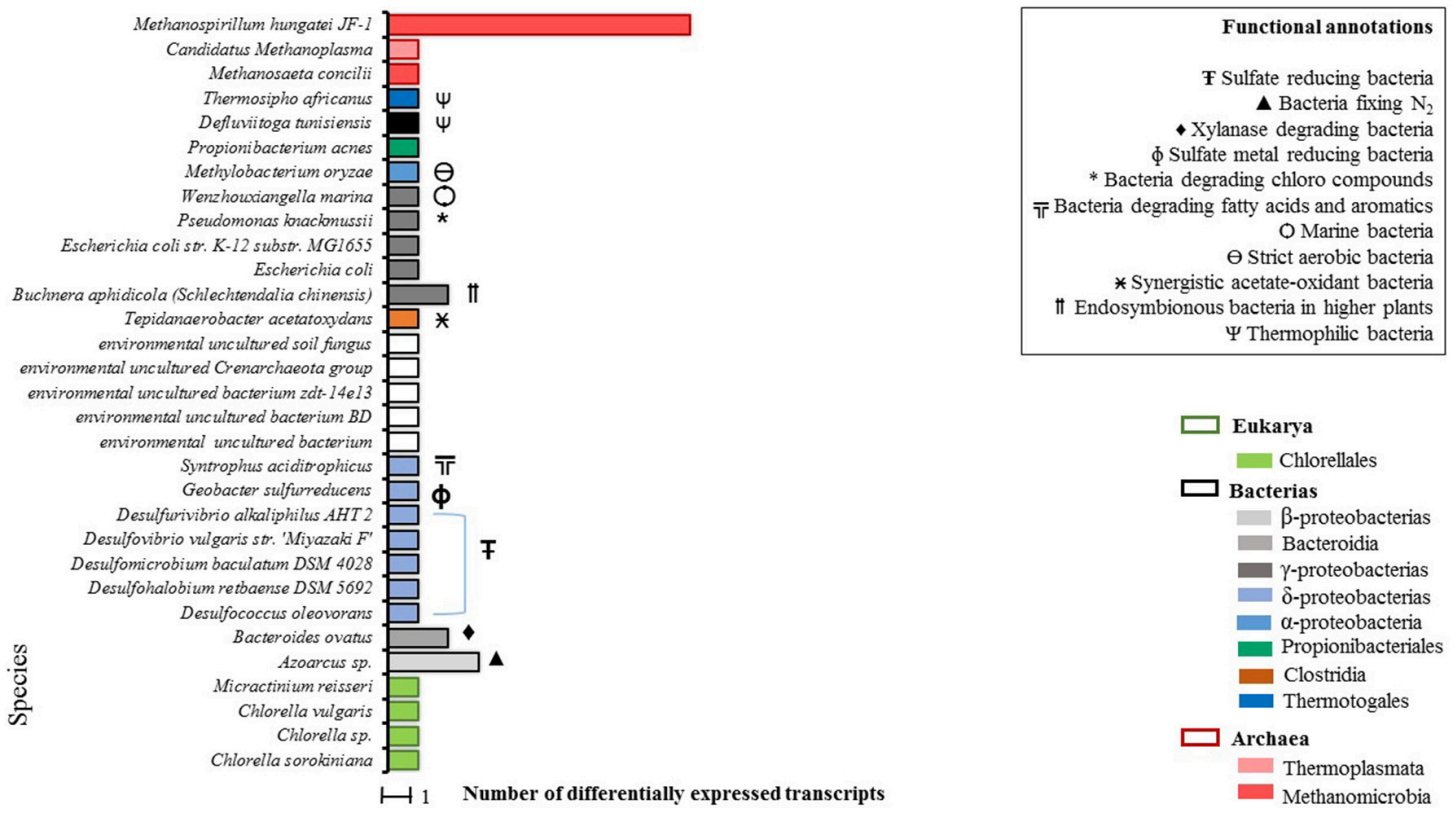
Total count of differentially expressed transcripts by species in WP samples (microalgal biomass without enzymatic pretreatment). Phylogenetic annotations are indicated at the level of classes (color-coded legend) as well as species functional annotations for transcripts with a taxonomic assignment (BLAST based; inset box). For blank horizontal bars, identification at the species level was not possible.

A total of 47 differentially expressed transcripts were identified for the condition WP (Figure 2), 39 of which were taxonomically annotated at the species level. For the remaining eight transcripts annotation at the species level was not possible.

From the taxonomic annotation of the 39 expressed transcripts in the WP condition, a predominant first cluster (C-I) that represents the 35% of the microbial community becomes evident. C-I is made up of sulfate-reducing bacteria. A second cluster (C-II) of extreme environment thermophilic bacteria was found, composed of *Thermosipho africanus, Defluviitoga tunisiensis* (which are closely related), and of *Wenzhouxiangella marina*. Additionally, three archaea species were detected in C-II: *Methanosaeta concilii* and *Methanospirillum hungatei* JF-1, which presented the highest quantity of annotated transcripts, belonging to the Methanomicrobia class and finally *Candidatus methanoplasma* of the Thermoplasmata class.

As expected, we got evidence of active microalgal cells which were represented by four species: *Chlorella* sp., *C. sorokiniana, Chlorella vulgaris*, and *Micractinium reisseri*, all belonging to the Chlorellaceae family.

#### Functionally active taxa in samples subjected to an enzymatic pretreatment

**Figure 3:**
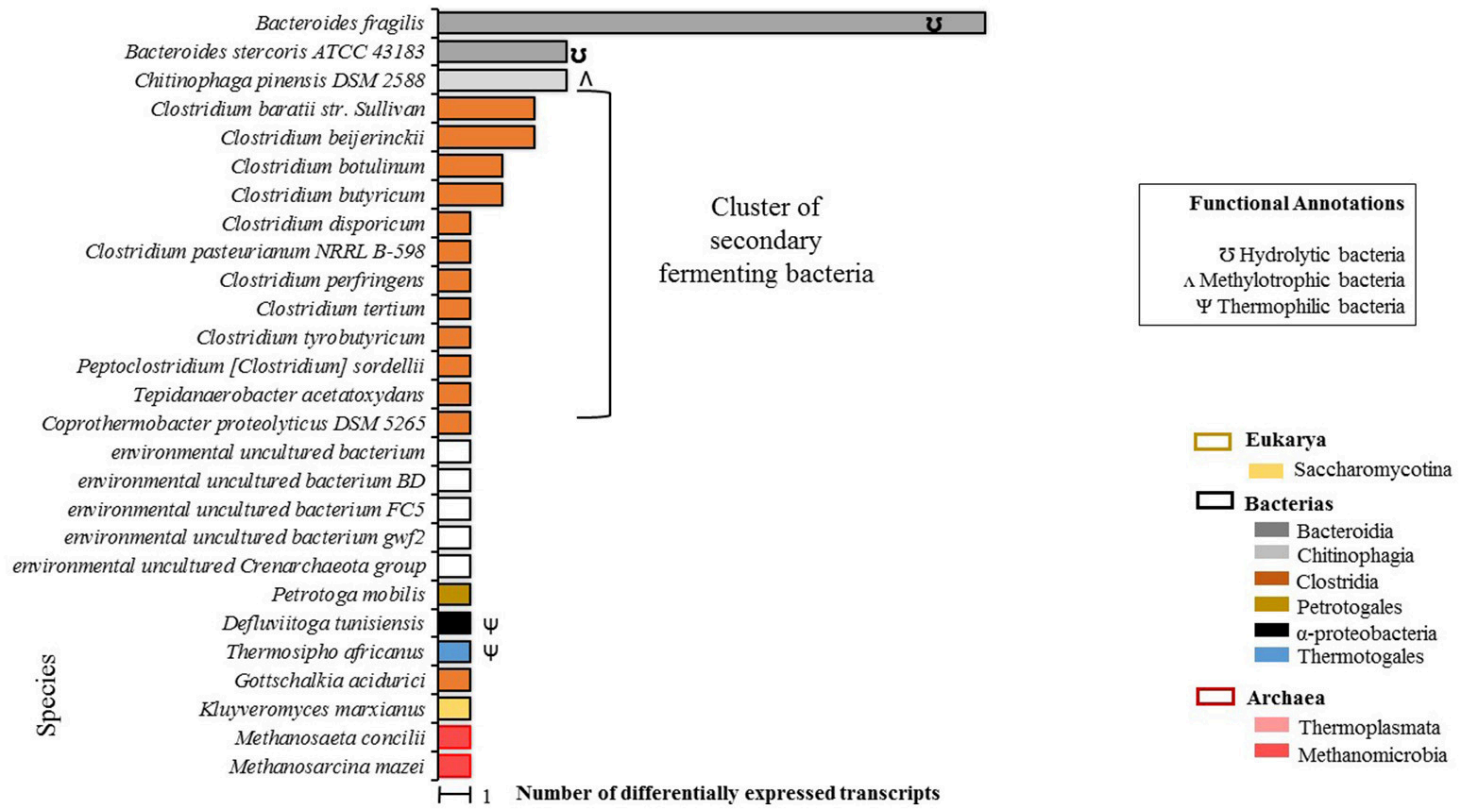
Total count of differentially expressed transcripts by species in EP samples (microalgal biomass with enzymatic pretreatment). Phylogenetic annotations are indicated at the level of classes (color-coded legend) as well as species functional annotations for transcripts with a taxonomic assignment (BLAST based; inset box). For blank horizontal bars, identification at the species level was not possible. Species belonging to secondary fermenting bacteria have been clustered together.

A total of 55 differentially expressed transcripts were identified in the EP condition, 50 of which were taxonomically annotated at the species level (Figure 3). Annotation at the species level was not possible for the remaining five transcripts.

A predominant bacterial cluster (C-I) was found which represented 50% of the microbial community. C-I was made up of secondary Clostridia-class fermentation bacteria. Additionally, a second cluster (C-II) of extreme environment thermophilic bacteria was foun composed of *T. africanus, Petrotoga molbilis*, and *D. tunisiensis* species, which represented the 14% of the microbial community. Two archaea species were detected: *M. concilii* and *Methanosarcina mazei*, both of the Methanomicrobia class. It should be noted that under this condition, no transcripts were identified for microalgae species, which could confirm that no living microalgal cells were actually present after the enzymatic pretreatment.

### Metabolic pathways activated across experimental conditions

#### Control samples

The number of active metabolic pathways identified for both conditions was low. However, we were able to map key enzymes of these metabolic pathways which provided strong evidence of the actual processes that were triggered in the microbial community in response to the experimental conditions being assayed. Table 2 shows the results of the prediction of key proteins/enzymes for the activation of metabolic pathways in the WP condition.

**Table 2:** Key proteins/enzymes prediction for metabolic pathways activation by enzyme mapping through the KEGG online platform of differentially expressed transcripts detected on WP samples.

**Protein/Enzyme**	**KEGG id**	**Organism from anaerobic sludge**	**E-value**	**KEGG pathway (id)**
Peptide/nickel transport system substrate-binding protein	K02035	*T. africanus*	6e^−50^	Quorum sensing (ko02014)
Peptidoglycan-associated lipoprotein	K03640	*Geobacter*	1e^−35^	Transporters (ko02000)
F-type H+-transporting ATPase subunit alpha	K02111	*S. aciditrophicus*	7e^−152^	Oxidative phosphorylation (ko00190)
Membrane fusion protein, multidrug efflux system	K03585	*G. sulfurreducens*	2e^−21^	beta-Lactam resistance (ko01501)
7,8-Dihydropterin-6-yl-methyl-4-(beta-D-ribofuranosyl)aminobenzene 5′-phosphate synthase	K06897	*D. vulgaris*	7e^−81^	Folate biosynthesis (ko00790)
DNA-binding protein HU-beta	K03530	Non-annotated	1e^−22^	DNA repair and recombination proteins (ko03400)
Cold shock protein (beta-ribbon, CspA family)	K03704	*G. sulfurreducens*	5e^−28^	Transcription factors (ko03000)
Large subunit ribosomal protein L4	K02926	*S. aciditrophicus*	6e^−100^	Ribosome (ko03010)
Archaeal flagellin FlaB	K07325	*M. hungatei*	2e^−82^	Secretion system (ko02040)
Beta-galactosidase	K01190	*E. coli*	8e^−33^	Glycan degradation (ko00511)
Branched-chain amino acid transport system substrate-binding protein	K01999	Non-annotated	4e^−52^	Quorum sensing (ko02014)
Prolyl-tRNA synthetase	K01881	Non-annotated	1e^−19^	Aminoacyl-tRNA biosynthesis (ko00970)
Butyryl-CoA dehydrogenase	K00248	*D. oleovorans*	4e^−85^	Fatty scid degradation (ko00071)
5,10-Methylenetetrahydromethanopterin reductase	K00320	*M. hungatei*	0.0	Methanogenesis, CO_2_ => methane (ko00680)
Alanine dehydrogenase	K00259	*P. acnes*	0.0	Alanine, aspartate and glutamate metabolism (ko00250)

Metabolic pathway activation was detected for processes involved in the processing of environmental information, carbohydrate and lipid metabolism, and in the activation of bacterial defense mechanisms. This shows some of the functional behavior of the microbial community which biomethanized a microalgal biomass with no damage or rupture to the cell wall. There was also evidence of bacterial *quorum sensing* (cellular process, ko02014). *Quorum sensing* detection could be indicative of genetic expression regulation in response to fluctuations in cell population density. Bacteria produce and release chemical signals (autoinducers) which become more concentrated as cell density increases (Waters and Bassler, 2005). *Quorum sensing* activation was detected in *T. africanus* (Bacteria, Thermotogae) and in *D. tunisiensis* (Bacteria, Petrotogae) both of which are phylogenetically related. The activation of *quorum sensing* was also detected in a third bacterium, although this could not be identified at the species level.

Bacterial organic material degradation was also detected. The activation of carbohydrate metabolism was recorded through galactose degradation (carbohydrate metabolism, ko00052) for *Escherichia coli* (Bacteria, γ-proteobacteria). In addition, lipid metabolism activation through fatty acids degradation by *Defluviitoga oleovarans* (Bacteria, δ-proteobacteria) and amino acids degradation (Metabolism of other amino acids ko 00430, ko00250) through the hydrolytic action of *Propionibacteria acnes* (bacteria, Propionibacteria) were also detected. Bacterial defense mechanisms activation was shown for *Geobacter sulfurreducens* (Bacteria, δ-proteobacteria) for which the synthesis of β-lactam of resistance was found (resistance to drugs, via β-lactam ko1501). For the archaea *M. hungatei* JF-1, methane metabolism activation was detected (ko, 00680) only for the pathway CO_2_ → CH_4_. Additionally, cell mobility activation for archae was apparently achieved through the flagellar protein pathway FlaB (ko02040). No prediction of proteins/enzymes was achieved that could indicate metabolic pathways activation in the microalgal species identified in the study.

#### Samples subjected to an enzymatic pretreatment

Some similarities were observed in EP samples with respect to WP samples mainly in relation to *quorum sensing* activation. However, differences with WP samples were observed for metabolic pathways involved in carbohydrates and lipids metabolism, as well as for the activated bacterial defense systems (Table 3).

**Table 3:** Key proteins/enzymes prediction for metabolic pathways activation by enzyme mapping through the KEGG online platform of differentially expressed transcripts detected on EP samples.

**Protein/Enzyme**	**KEGG id**	**Organism from anaerobic sludge**	**E-value**	**KEGG pathway (id)**
Small acid-soluble spore protein D	K06421	*C. butyricum*	1e^−28^	Unknown
Thiol peroxidase, atypical 2-Cys peroxiredoxin	K11065	*C. beijerinckii*	5e^−30^	Oxidoreductases (ko01000)
Glycerol-3-phosphate acyltransferase PlsX	K03621	*D. tunisiensis*	0.0	Glycerolipid metabolism (ko00561)
ATP-binding protein involved in chromosome partitioning	K03593	*D. tunisiensis*	0.0	Mitochondrial biogenesis (ko03029)
Small acid-soluble spore protein D	K06421	*C. perfringens*	1e^−30^	Unknown
Putative ABC transport system ATP-binding protein	K05833	*D. tunisiensis*	3e^−160^	Transporters (ko02000)
Beta-glucosidase	K05349	*D. tunisiensis*	0.0	Starch and sucrose metabolism (ko00500)
Putative transposase	K07497	*W. marina*	6e^−43^	Unknown
UDP-N-acetylglucosamine 2-epimerase	K01791	*P. mobilis*	7e^−71^	Amino sugar and nucleotide sugar metabolism (ko00520)
Alpha-amylase	K01176	*D. tunisiensis*	3e^−127^	Starch and sucrose metabolism (ko00500)
Putative ABC transport system substrate-binding protein	K01989	*D. tunisiensis*	0.0	Transporters (ko02000)
Phosphopantothenoylcysteine decarboxylase/phosphopantothenate cysteine ligase	K13038	*D. tunisiensis*	3e^−38^	Pantothenate and CoA biosynthesis (ko00770)
Tyrosine-protein kinase Etk/Wzc	K16692	*D. tunisiensis*	0.0	Protein kinases (ko01001)
5-Methylcytosine-specific restriction enzyme B	K07452	*D. tunisiensis*	0.0	Prokaryotic defense system (ko02048)
HSP20 family protein	K13993	*C. pasteurianum*	2e^−32^	Chaperones and folding catalysis (ko03110)
Branched-chain amino acid transport system permease protein	K01998	*P. mobilis*	1e^−139^	Quorum sensing (ko02024)
Flagellin	K02406	*C. pasteurianum*	2e^−104^	Flafellar assembly (ko02040)
Peptide/nickel transport system substrate-binding protein	K02035	*T. africanus*	6e^−50^	Quorum sensing (ko02024)

Regarding bacterial *quorum sensing* activation (cell process, ko02014), it was identified for *T. africanus* and *P. mobilis* (Bacteria, Petrotogae). It should be noted that for the WP condition, *quorum sensing* activation was also recorded for *T. africanus* and for a Petrotogae bacteria (*D. tunisiensis*). Regarding organic matter degradation, we collected evidence supporting that carbohydrate metabolism activation was through the degradation pathway of starch and sucrose (carbohydrate metabolism, ko00050) for *D. tunisiensis* (Bacteria, Petrotogae). For the same bacteria, co-factor metabolism activation was through the Pantothenate pathway and CoA biosynthesis (ko00770). Through this pathway cofactors play a key role in the biosynthesis and decomposition of fatty acids as well as in the biosynthesis of polyketides (secondary metabolites) and non-ribosomal peptides (Begley et al., 2001). Amino sugars degradation pathway and nucleotide sugar metabolism (ko00520) was also identified in *P. mobilis* (Bacteria, Petrotogae).

Like in the WP condition, the activation of bacteria defense mechanisms was recorded in the EP condition. In this case the activation of the defense mechanism was via specific restriction enzymes and hydrolytic enzymes (ko02048) identified in *D. tunisiensis*. For the same species, there was evidence of environmental information processing, via the ABC membrane transport pathway, which connects the ATP hydrolysis to the active transport of a wide variety of substrates such as ions, sugars, lipids, sterols, peptides, proteins (ko 02010). No prediction of proteins/enzymes was achieved that could indicate metabolic pathways activation in the archaea species detected under this condition.

## Discussion

Functional changes observed in microbial communities under both experimental conditions provide insights on how the community of both bacteria and archaea “restructures” based on whether or not an enzymatic pretreatment is performed. Such changes could mediate the community response to a new configuration of microalgal biomass. *De novo* metatranscriptome analysis of WP and EP turned out to be useful in providing a high resolution “picture” of the microbial community genetic expression without the need of an *a priori* knowledge of the genomes present (Moran, 2009; Vanwonterghem et al., 2014).

We collected phenotypic (methane production) a molecular (genetic expression) information suggesting that the anaerobic sludge associated microbial community managed to adapt to new configuration of organic matter that arose from the enzymatic pretreatment of the microalgal biomass. Changes in the configuration of organic matter when applying pretreatments to microalgal biomasses have been reported by Jiang et al. (2011). These authors applied pretreatments to the microalgal biomass with ultrasound and with the MFC (Microbial Fuel Cell) technique. After the pretreatments they analyzed the components being degraded by the bacterial fraction associated to an anaerobic sludge. They concluded that the components degraded differed among the pretreatments. For the pretreatment with ultrasound, mainly aromatic proteins were solubilized, while for the pretreatment with the MCF technique carbohydrates were the main degraded substrates (Jiang et al., 2011). This situation can be explained by the fact that extracellular polymeric substances (EPS) is one of the main constituents of the total microalgal organic matter (Mishra and Jha, 2009). EPS are metabolic products that can be released to the extracellular medium and/or may be accumulated on the cell surface, providing protection to cells against a hostile environment. Therefore, once the pretreatment was applied, the microalgal cells were able to release different components depending on the type of pretreatment, thus changing the bioavailability of organic matter for bacteria.

A large number of reads could not be assigned to known sequences, which made it hard to obtain a complete transcript analysis. This is due to the large quantity of bacteria and archaea found in association to anaerobic sludge that have not yet being sequenced and/or annotated. Therefore, the conclusions we provide in the following paragraphs must still be taken with caution since they arose from a still incomplete meta-transcriptome. Despite this, the present study is a pioneering effort to shed light on the main changes that might occur at the metatranscriptomics level in the context of biomethanization and biomass pretreatment, when little a priori genomic information of the target microbial community is available.

Changes observed in the metatranscriptome of the studied microbial community could have been caused by multiple factors, for example, a change in bacteria energy source or in microalgal defense mechanisms activation, which brought about new interactions among microorganisms (Bochner, 2009). The following sections discuss some of these factors.

### Energy sources

In WP samples microalgal biomass comprised living cells which suggests little to no damage of the cell wall. In contrast, in EP samples microalgal cells were mostly dead in their vast majority due to significant cell wall damage. This could have determined the type of organic matter bacteria were assimilating in both samples. Analysis of active metabolic pathways suggests that in WP samples bacteria energy sources came mainly from sugar-based organic matter degradation, such as galactose, fatty acids, and some amino acids. Under this condition, hydrolytic bacteria populations must degrade a rigid cell wall. Activated enzymes found in WP samples provided evidence for fatty acids (Acyl-CoA dehydrogenase), carbohydrates (Beta galactosidase), and amino acids (Alanine dehydrogenase) degradation.

The activity of the beta-galactosidase enzyme (k01190) is fundamental for galactose metabolism which is commonly activated in conjunction to amino sugars and nucleotide sugars metabolism. Such activations are common in hydrolytic bacteria metabolizing for instance glucosamine, which has been reported as the main component of the rigid cell wall of the microalgae species *C. vulgaris* and *C. sorokiniana* (Takeda, 1991; Templeton et al., 2012).

In EP samples, bacteria energy sources seemed to come from sugary organic matter degradation such as sucrose and starch. We got evidence for the activation in *D. tunisiensis* of the cytoplasmic α-amilase, an enzyme of the starch hydrolase family that is key for the degradation of this compound (Janecek, 1997). Starch has been reported as an intracellular element, which shows that in EP samples energy came from intracellular organic components freed to the medium as consequence of cell wall breakdown.

### Dominant taxa, key functional roles in microbial structure

The microbial community in WP samples appeared to be dominated by an active fraction of γ- and δ-proteobacteria. The δ-proteobacteria group is full of sulfate reducing bacteria (SRB). SRB are anaerobic microorganisms that use sulfate as acceptor of terminal electrons from the degradation of organic compounds with the concomitant production of H_2_S (Muyzer and Stams, 2008). SRB digest fermentation products such as acetate, butyrate, lactate and hydrogen (Gerardi, 2003). In EP samples, the microbial community was widely dominated by a large variety of Clostridium species (13 species). Bacteria from the Clostridium genus are characterized by their intensive fermentative metabolism. They can use numerous organic compounds as carbon and nitrogen sources. Based on the presence of this fermenting bacteria cluster of *Clostridium* genus, we can infer that the new organic matter configuration that resulted from biomass enzymatic pretreatment, provided favorable conditions for the growth of Clostridium species. It has been reported that Clostridium species can exhibit an opportunistic behavior i.e., high adaptability, when an increase in soluble organic matter in the medium is verified (Lee et al., 2008; Szymanowska-Powałowska et al., 2014).

### Ecological interactions

In both samples (WP and EP), transcripts were found that codify for enzymes associated with bacteria quorum sensing (QS 02024). Quorum sensing is a regulator system that allows bacteria to share information about cell density and adjust their genetic expression in relation to their interaction with the environment (Williams, 2017). Solely based on transcript analysis, it is not possible to determine the causes of QS activation, that is, if it was due to bacteria-bacteria or microalga-bacteria defense mechanisms. However, we can mention that some of the processes controlled by QS include virulence, competition, conjugation, antibiotic production, motility, sporulation, and biofilm formation as bacteria defense mechanisms (Waters and Bassler, 2005). In the case of bacteria-bacteria interactions, the defense mechanisms imply a cell-to-cell communication that leads to the expression and release of bioactive substances to the surroundings and that influence the behavior of other microorganisms found in the environment (Waters and Bassler, 2005). In the case of bacteria-microalga mechanisms, QS allows bacteria to detect microalgal cells. The detection signal is precise and regulated according to the microalga, its growth stage and biomass density (Mitsutani et al., 2001). QS acts as a bacteria inducer to produce and secrete “algicide” substances in the surrounding medium (Waters and Bassler, 2005). Microalgae are able to secrete compounds that imitate the QS detection signals of many Gram-negative bacteria, resulting in stimulant, or inhibitor effects. For example, Shehata et al. (2013) described the effect of *Chlorella vulgaris* on the growth of different Clostridium species. This might be the reason why in WP samples where Chlorella cells were still alive, no proliferation of Clostridium species was detected. The opposite was observed in EP samples where Chlorella living cells were wiped out.

### Methanogenesis

The final biochemical phase of the anaerobic digestion process is methanogenesis. During methanogenesis methane is metabolized by methanogenetic archaea in the global carbon cycle (Lee et al., 2009). In this study, differences were observed in the abundance and diversity of methanogenetic archaea across experimental conditions. In WP samples three archaea species were detected: *C. methanoplasma, M. concilii* (with a very low number of transcripts), and *M. hungatei* JF-1 (with a high number of transcripts). We also recorded the activation of the methanogenic pathway that uses CO_2_ as substrate in the WP samples.

For *M. hungatei*, syntrophic relations with other microorganisms have been described (Walker et al., 2012). A syntrophic relationship is a specific form of microbial mutualism that occurs between acetogenic bacteria or BSRs and archaea, which can use organic or inorganic substances as substrates for fermentation. In these associations, BSRs as *Desulfovibrio* species, act as secondary fermenters that are obligatorily bound by interspecific electrons to the metabolic activity of methanogenetic archaea (Kato and Watanabe, 2010). A syntrophic relationship between *M. hungatei* and BSRs would explain the large quantity of transcripts identified for this archaea in the WP samples.

Two pathways have been described for methane formation from acetate as substrate. The first is the acetoclastic pathway, carried out by Methanosarcinaceae or Methanosaetaceae. The second pathway involves a two-stage reaction in which acetate is first oxidized to H_2_ and CO_2_ which are then converted to methane. This reaction is performed by acetate oxidizing bacteria, such as the Clostridium species found in the EP samples in a syntrophic association with hydrogenotrophic methanogens (Methanomicrobia or Methanobacteria) (Karakashev et al., 2006).

**Figure 4:**
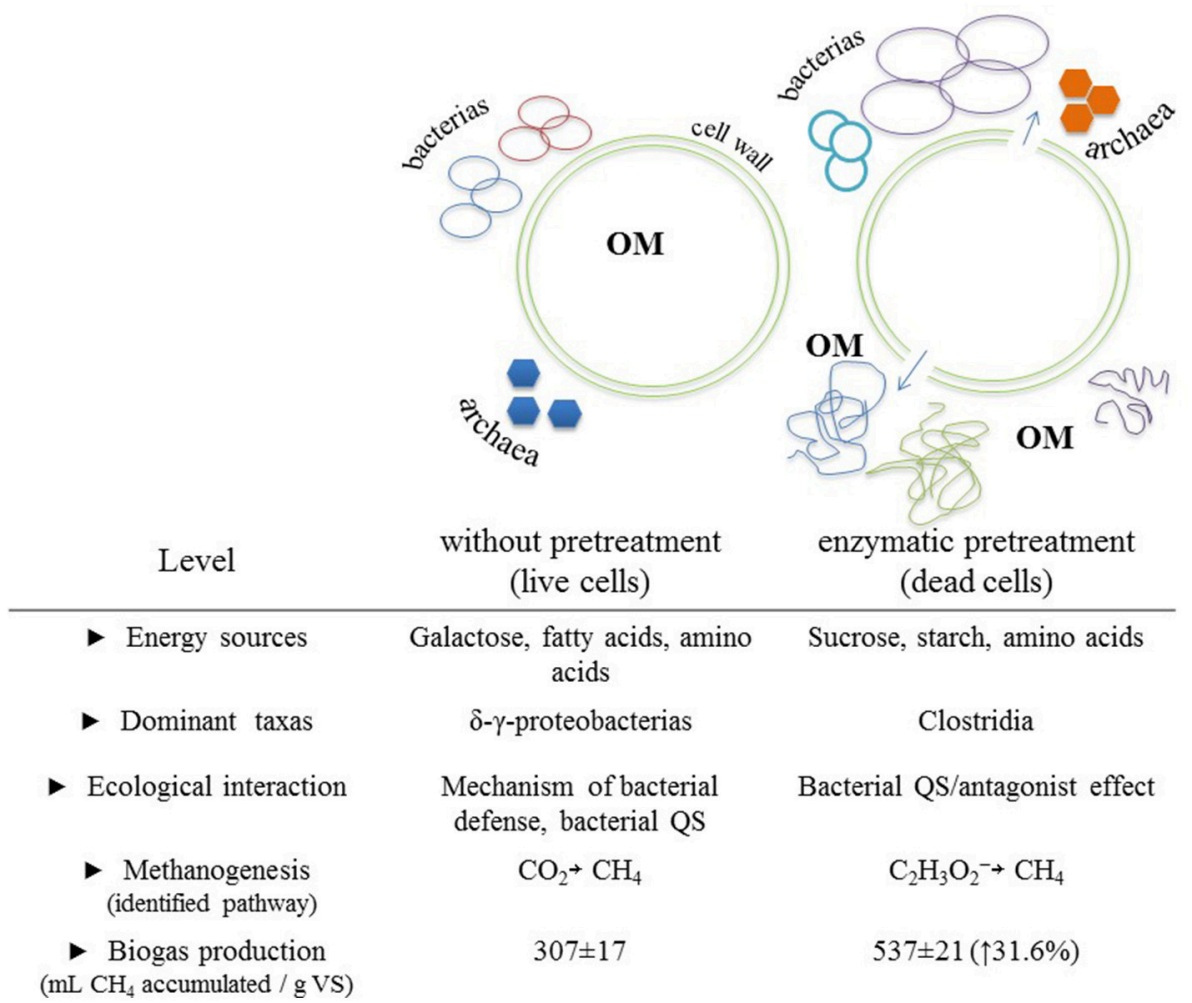
Main differences observed between the biomethanization process of a microalgal biomass with and without enzymatic pretreatment. Differences were categorized into five levels. Cartoonish representations of biomethanization process with emphasis on the expected changes of OM configurations between both experimental conditions are included. OM, organic matter.

Finally, it can be mentioned that the molecular tools used in this study allowed us to link activated metabolic pathways to a diversity of prokaryotes under two different experimental conditions that differed mainly in available energy sources, dominant taxa, ecological interactions, and metabolic pathways for methanogenesis. Figure 4 shows a general scheme depicting the main differences found across experimental conditions and that we believe are directly linked to the applied enzymatic pretreatment.

## Author contributions

OC conceived the study, designed, and performed the experiments, evaluated the data, and drafted the manuscript. AS-R performed bioinformatics analyzes and drafted the manuscript. RC and LG supervised the work and assisted in drafting the manuscript. All authors read and approved the final manuscript.

### Conflict of interest statement

The authors declare that the research was conducted in the absence of any commercial or financial relationships that could be construed as a potential conflict of interest.
